# Mineralocorticoid receptors are implicated in the initial steps of the cardiorenal damage induced by ethanol

**DOI:** 10.3389/fcvm.2026.1855068

**Published:** 2026-06-23

**Authors:** Thales M. H. Dourado, Gustavo F. Pimenta, Barbara M. Marchetti, Alessandra O. Silva, Carlos R. Tirapelli

**Affiliations:** 1 Programa de Pós-Graduação em Farmacologia, Faculdade de Medicina de Ribeirão Preto, Universidade de São Paulo (USP), Ribeirão Preto/São Paulo, Brazil; 2 Departamento de Ciências BioMoleculares, Faculdade de Ciências Farmacêuticas de Ribeirão Preto, USP, Ribeirão Preto/São Paulo, Brazil

**Keywords:** alcohol, aldosterone, heart, kidney, oxidative stress

## Abstract

**Background:**

Oxidative stress is an early mediator of ethanol–induced cardiorenal injury and dysfunction. The enzyme nicotinamide adenine dinucleotide phosphate (NADPH) oxidase plays a key role in the generation of reactive oxygen species (ROS) triggered by ethanol. In certain tissues, the pro–oxidative and pro–inflammatory effects of ethanol occur via activation of mineralocorticoid receptors (MR). We hypothesized that MR blockade would prevent the redox imbalance induced by ethanol during the early stages of cardiac and renal changes.

**Methods:**

Male Wistar Hannover rats were treated with ethanol for five weeks. The contribution of the MR pathway to the cardiorenal effects of ethanol were evaluated with potassium canrenoate, an antagonist of the MR (MRA).

**Results:**

Blockade of MR prevented the increase in lipoperoxidation and superoxide (O_2_•^−^) levels in the left ventricle and renal cortex of ethanol-treated rats. Increased concentrations of hydrogen peroxide (H_2_O_2_) were detected in the renal cortex, but not in the left ventricle of ethanol-treated rats, and treatment with the MRA prevented this response. Ethanol augmented cardiac levels of thromboxane (TX)B_2_, a stable metabolite of TXA_2_ and the renal concentration of prostaglandin (PG)E_2_. These responses were abrogated by MR blockade.

**Conclusion:**

The MR pathway mediates pro-oxidative effects in the early stages of ethanol-induced cardiorenal dysfunction.

## Introduction

Oxidative stress is a critical step that initiates the array of the adverse changes induced by ethanol in the cardiorenal system. In the heart and kidney, ethanol favors the generation of superoxide (O_2_•^−^), a free radical produced by the enzyme nicotinamide adenine dinucleotide phosphate (NADPH) oxidase (1, 2). Upregulation of NADPH oxidase occurs in the heart and kidney after ethanol consumption, and this response is linked to the overproduction of reactive oxygen species (ROS) (1, 3–5). As a consequence of ROS generation, lipoperoxidation occurs in the cardiorenal system (6, 7). Ethanol-induced redox imbalance also results from its ability of affecting enzymatic and nonenzymatic antioxidant systems in the heart and kidney (8, 9).

The renin-angiotensin-aldosterone system (RAAS) is an important mediator of the effects of ethanol in the cardiorenal system (10). Ethanol-induced RAAS activation leads to increased levels of circulating aldosterone (11, 12). The latter mediates some of the effects of ethanol in distinctive tissues through mineralocorticoid receptors (MR). Blockade of these receptors prevents the pro-oxidative and pro-inflammatory effects of ethanol (13–16). Despite the well-known contribution of aldosterone/MR signaling to cardiac and renal damage in pathophysiological conditions, its involvement in the cardiorenal dysfunction induced by ethanol remains unclear.

Knowing that aldosterone/MR signaling favors oxidative stress in pathological conditions and that it underlies the deleterious effects of ethanol in some tissues, we hypothesized that blockade of MR would prevent ethanol-induced redox imbalance in the early stages of ethanol-induced cardiac and renal changes. Here, we sought to evaluate the involvement of MR in the redox imbalance that occurs during the early stages of the cardiac and renal toxicity induced by ethanol.

## Methods

### Animals and grouping

The cardiac and renal effects of ethanol were evaluated in male Wistar *Hannover* rats 50–60 days old (weighing between 230 and 260 g). With this purpose, rats were exposed to ethanol for five weeks as previously described (12, 13). In brief, ethanol-treated rats were submitted to a period of adaptation of two weeks in which they that free access to solutions of ethanol 5%, in the first week, and 10% in the second week (all in volume ratios). Then, rats had free access to ethanol 20% for three weeks. Blood ethanol levels in the range of 40–50 mmol/L are achieved in rats using this protocol of ethanol feeding (17). This concentration is within the one found in heavy drinkers (18). This protocol leads to decreases in body mass, food consumption, and fluid intake in rats from the ethanol group compared to control rats. Additionally, ethanol-treated rats showed increased circulating levels of renin, angiotensin I, angiotensin II, and aldosterone, confirming activation of the RAAS (12, 13, 15, 19).

The contribution of MR to the effects of ethanol in the heart and kidney were assed with potassium canrenoate (Cat. No. C7287, Sigma-Aldrich, St. Louis, MO, USA), an antagonist of MR (MRA). Potassium canrenoate (30 mg/kg/day) was dissolved in saline and administered by gavage (20). Rats from the control and MRA groups (not exposed to ethanol) had free access to commercial rat lab chow and filtered water *ad libitum*.

At the end of the experimental stage (5th week), rats were anesthetized intraperitonially with urethane (1.25 g/kg, administered as a 25% solution; 5 mL/kg; Cat. No. 51-79-6, Sigma-Aldrich, St. Louis, MO, USA), and then euthanized by exsanguination followed by diaphragm rupture. The left ventricle and the renal cortex were isolated and storage at −80 ^o^C.

### Determination of serum levels of creatinine and creatine phosphokinase (CK)-MB activity

Syringes containing heparin were used to collect blood from the inferior vena cava. Blood samples were centrifuged (6,500 × *g*, 10 min, 4 °C). Then, 12.5 and 7.5 µL of serum were used for the determination of creatinine levels (at 510 nm) and CK-MB activity (at 340 nm), respectively. The assays were conducted following instructions of commercial kits (Cat. No. 35 and 118, Labtest Diagnostica, Lagoa Santa, MG, Brazil). Results are given as mg/dL for creatinine and U/L for CK-MB activity.

### Determination of ROS levels and lipoperoxidation

A chemiluminescent assay was used to determine the generation of O_2_•^−^ as previously described (20). In brief, the luminescent probe lucigenin (bis-N-methylacridinium nitrate; Cat. No. 2315-97-1, Sigma-Aldrich) was added to the samples. A luminometer was used to detect the luminesce before and after the addition of NADPH (Cat. No. 2646-71-1, Sigma-Aldrich), a substrate of the enzyme NADPH oxidase. Results are expressed as relative light units (RLU) per mg of protein. The concentration of H_2_O_2_ was determined using a fluorogenic substrate (Cat. No. A22188, Amplex®Red, Invitrogen, Carlsbad, CA, USA). The assay was conducted as previously described (21). Results are given as nmol per mg protein.

The concentration of malondialdehyde (MDA) was determined as previously described (13). Briefly, the tissues were homogenised, centrifuged (1,600 × *g*, 10 min, 4 °C), and the supernatant was used to determine MDA concentration colorimetrically (at 532 nm). Results are given as mmol/L per mg of protein.

In all protocols, protein concentration was measured using the Lowry assay (Bio-Rad Laboratories, Hercules, CA, EUA).

Determination of SOD, catalase and glutathione peroxidase (GPx) activities and reduced glutathione (GSH) concentration.

Total SOD activity was determined in homogenates of the left ventricle and renal cortex. The tissues were homogenized in PBS. The homogenates were centrifuged (10,000 × *g*, 10 min, 4 °C), and then the supernatant was used to determine SOD activity following instructions of a commercially available kit (Cat. No. 19160, Sigma-Aldrich). SOD activity was determined at 450 nm, and results are given as percentage (%) inhibition per mg protein.

Catalase activity was determined by measuring the consumption of H_2_O_2_ as previously described (22). In brief, the tissues were homogenized in phosphate buffer and centrifuged (10,000 × *g*, 10 min, 4 °C). The supernatant was transferred to quartz cuvettes. Then, reaction buffer was added to the cuvettes. Absorbance was measured (at 240 nm) during 1 min. The results are expressed in unit (U) per mg protein. One U was defined as the amount of enzyme need to decompose 1 mmol/L of H_2_O_2_ within one min.

The assay used to determine GPx activity is based on the protocol previously published (22). Briefly, tissues were homogenized in PBS, centrifuged (5,000 × *g*, 10 min, 4 °C), and then the supernatant was incubated (5 min) at room temperature with reduced glutathione, PBS, and NADPH. Tert-butylhydroperoxide was added to the tubes and the absorbance was measured (at 340 nm) during 6 min. The results are expressed in nmol/min per mg of protein.

The concentration of GSH was determined colorimetrically as previously described (23). The assay is based on the reaction of GSH by s5,5′-dithio-bis-(2-nitrobenzoic acid) (DTNB) with GSH to form 5′-thio-2-nitrobenzoic (TNB). The latter is a yellow product that is detect at 415 nm. Results are expressed as µg of GSH per mg of protein.

### Enzyme-linked immunosorbent assay (ELISA)

The tissues were homogenized in PBS, centrifuged (6,500 × *g,* 10 min, 4° C) and the supernatant was used to determine TXB_2_, PGE_2_, IL-6 and IL-1β concentrations using commercially available kits (Cat. No. 501020 and 514010, Cayman Chemical Ann Harbor, MI, USA; Cat. No. RAB0312 and RAB 0278, Sigma-Aldrich). Results are given as pg per mg of protein.

### Determination of myeloperoxidase (MPO) and N-acetyl-beta-D-glucosaminidase (NAG) activities

Determination of MPO and NAG activities were conducted as previously described (2). In brief, tissues were homogenized in ice-cold PBS and then centrifuged (9,600 × *g*, 10 min, 4 °C). The pellet was lysed with NaCl (0.2%) and then an equal volume of a solution containing NaCl and glucose was added. After centrifugation (9,600 × *g*, 10 min, 4 °C), both 3,3′-5,5′-tetramethylbenzidine and H_2_O_2_ were added to the supernatant to determine MPO activity (at 450 nm). For determination of NAG activity, samples were lysed with NaCl (0.2%), centrifuged (9,600 × *g*, 10 min, 4 °C) and then the pellet was resuspended in ice-cold saline (Triton X-100 0.1%) and re-homogenized. The homogenates were centrifuged, and the supernatants were incubated with p-nitrophenyl-N-acetyl-β-D-glucosaminide and citrate buffer. Glycine buffer was used to stop the reaction and absorbance was determined at 405 nm. Results were given as relative units (RU) of optical density (O.D.) per mg of protein.

### Statistical analysis

Results are presented as means ± standard error of the mean (S.E.M.). Two-way analysis of variance (ANOVA) followed by Tukey's comparison test was used to analyse the data using the software GraphPad® Prism 8.02 (GraphPad Software Inc.). In all cases, values of *p* < 0.05 were considered significant.

## Results

**Figure 1:**
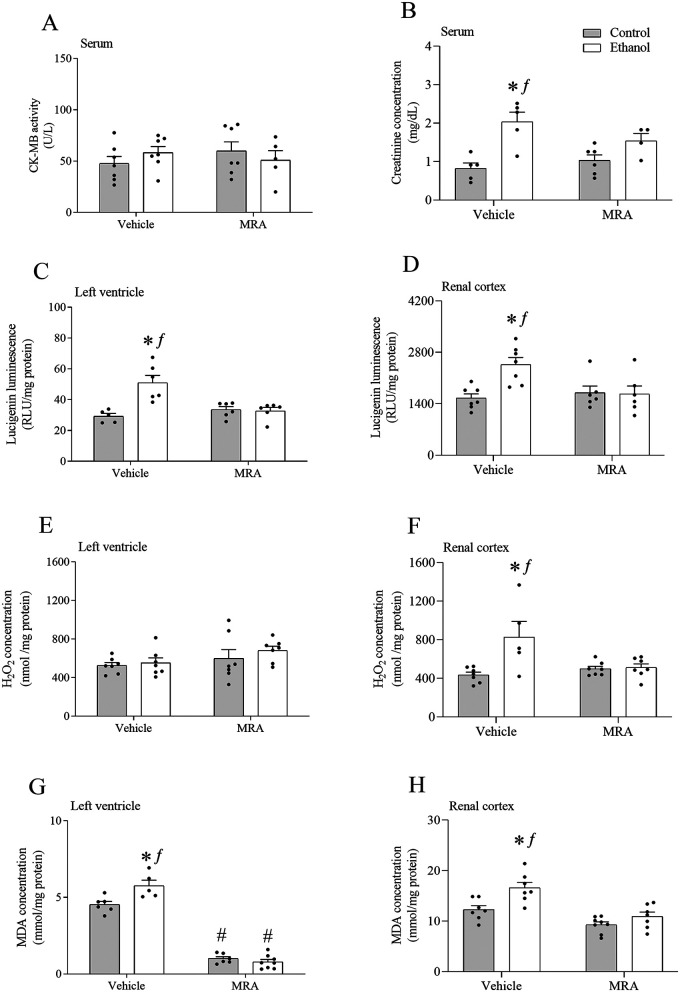
Role of mineralocorticoid receptors (MR) on reactive oxygen species (ROS) generation and lipoperoxidation in the left ventricle and the renal cortex of ethanol-treated rats. Serum levels of creatine phosphokinase (CK)-MB **(A)** and creatinine **(B)** were determined colorimetrically as biomarkers of cardiac and renal dysfunction, respectively. NADPH oxidase-induced superoxide (O_2_•^−^) generation was determined using the lucigenin chemiluminescence assay **(C,D)**. The concentration of hydrogen peroxide (H_2_O_2_) was measured using a fluorogenic substrate **(E,F)**. The levels of malondialdehyde (MDA) were determined colorimetrically as an estimative of lipoperoxidation **(G,H)**. Control, rats receiving vehicle treatment; Ethanol, rats exposed to ethanol; MRA, rats treated with mineralocorticoid receptor antagonist; Ethanol + MRA, rats co-treated with ethanol and MRA. Results are expressed as mean ± standard error of the mean (SEM), with n = 4-7 animals per group. Statistical analysis, Two-way ANOVA followed by Tukey's *post hoc* test. *P < 0.05 vs. control group; ^ƒ^*P* < 0.05 vs. MRA and ethanol-MRA groups; ^#^*P* < 0.05 vs. control and ethanol groups.

Our first step was to determine whether ethanol promotes tissue dysfunction. To this end, we measured biomarkers of cardiac and renal dysfunction. Neither ethanol nor the MRA affected the serum activity of CK-MB (Figure 1A). Ethanol consumption increased the serum levels of creatinine, and this response was prevented by the MRA (Figure 1B). Given that oxidative stress is a key mediator of the harmful effects of ethanol, we next assessed cardiac and renal ROS generation and lipid peroxidation. Increased levels of O_2_•^−^ were detected in the left ventricle and renal cortex of ethanol-treated rats. In both cases, blockade of MR prevented this response (Figures 1C,D). In the left ventricle, no changes were detected in the levels of H_2_O_2_ after treatment with ethanol or the MRA (Figure 1E). However, ethanol consumption increased H_2_O_2_ concentrations in the renal cortex, a response that was prevented by the MRA (Figure 1F). The left ventricle and the renal cortex of ethanol-treated rats showed increased concentrations of MDA, and, in both cases, blockade of MR prevented this response (Figures 1G,H). Of note, the MRA had an effect *per se* on the concentration of MDA in the left ventricle (Figure 1G). Thus, MR mediate ROS generation and lipid peroxidation in response to ethanol consumption, which acts in a tissue-specific manner.

**Figure 2:**
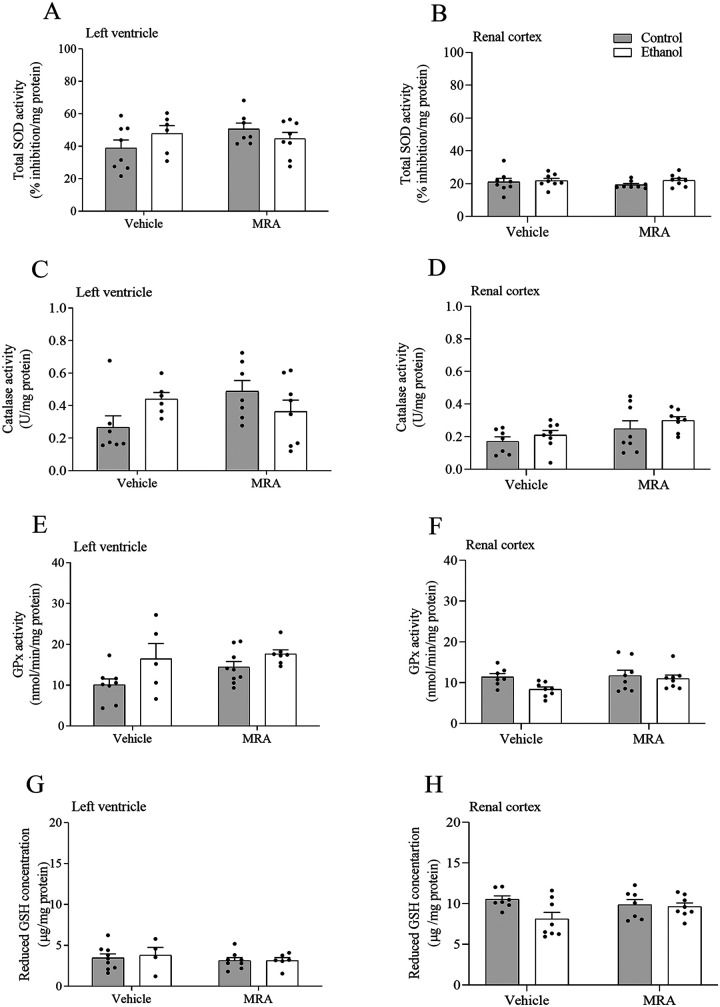
Enzymatic and non-enzymatic antioxidant status in the left ventricle and renal cortex of ethanol-treated rats. The activities of superoxide dismutase (SOD) **(A– B)**, catalase **(C,D)**, and glutathione peroxidase (GPx) **(E,F)** were determined colorimetrically as markers of the enzymatic antioxidant system. The concentration of reduced glutathione (GSH) **(G,H)** was measured as an indicator of the non-enzymatic antioxidant system. Control, rats receiving vehicle treatment; Ethanol, rats exposed to ethanol; MRA, rats treated with mineralocorticoid receptor antagonist; Ethanol + MRA, rats co-treated with ethanol and MRA. Results are expressed as mean ± standard error of the mean (SEM), with *n* = 4–8 animals per group. Statistical analysis, Two-way ANOVA followed by Tukey's *post hoc* test.

The redox imbalance induced by ethanol may also result from a reduced antioxidant capacity. To investigate this possibility, both enzymatic and non-enzymatic antioxidants were assessed. Neither ethanol nor the MRA affected SOD activity in the left ventricle or the renal cortex (Figures 2A,B). Similarly, no changes in catalase (Figures 2C,D) or GPx activities (Figures 2E,F) were found in the left ventricle or the renal cortex after treatment with ethanol or the MRA. Treatment with ethanol or the MRA did not affect the concentration of reduced GSH in the left ventricle or the renal cortex (Figures 2G,H). Overall, these findings indicate that ethanol did not affect antioxidant capacity in the heart and/or kidney.

**Figure 3:**
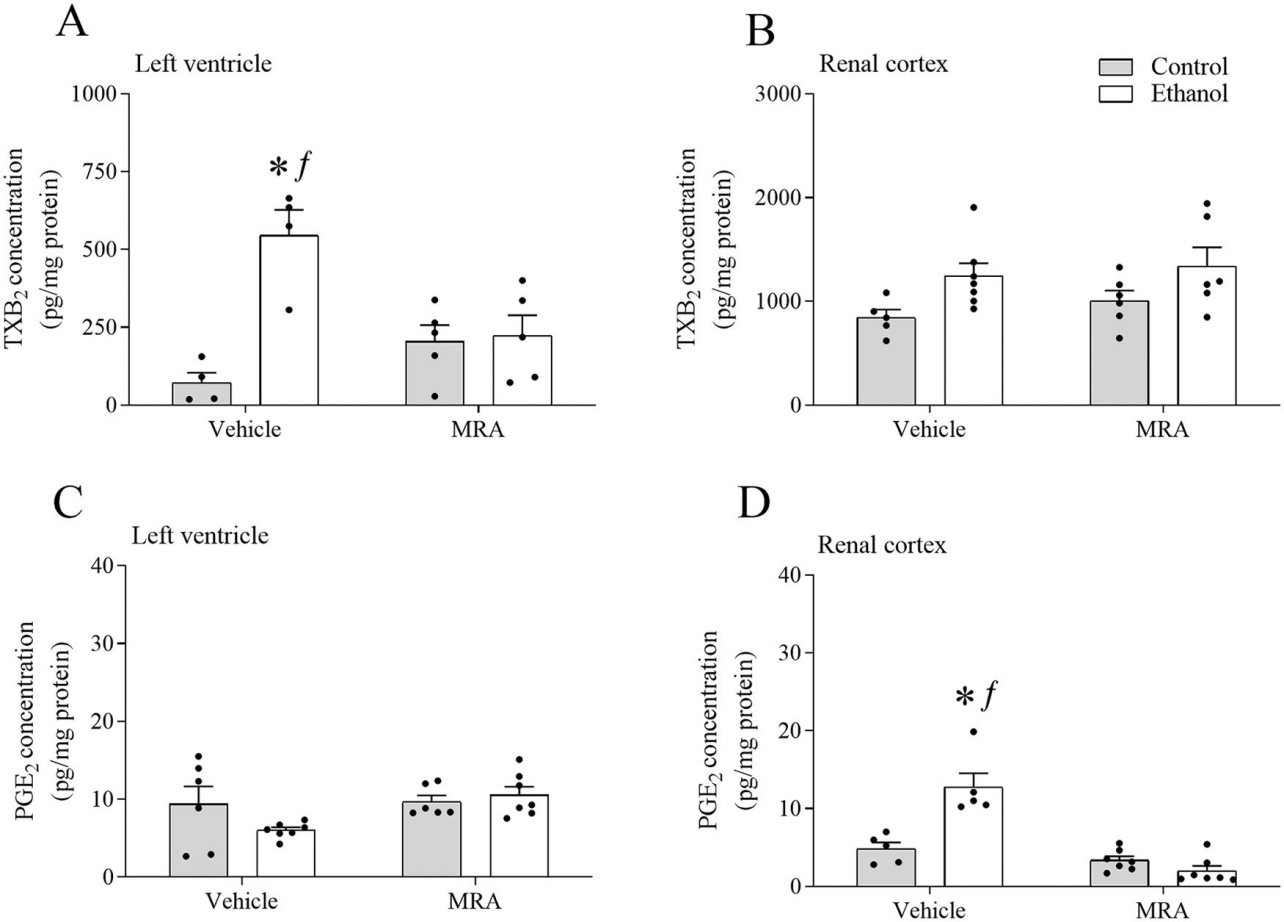
Role of mineralocorticoid receptors (MR) on eicosanoids production in the left ventricle and the renal cortex of ethanol-treated rats. The concentrations of thromboxane (TX)B_2_ **(A,B)**, a stable metabolite of TXA_2_, and prostaglandin (PG)E_2_ **(C,D)** were determined by ELISA. Control, rats receiving vehicle treatment; Ethanol, rats exposed to ethanol; MRA, rats treated with mineralocorticoid receptor antagonist; Ethanol + MRA, rats co-treated with ethanol and MRA. Results are expressed as mean ± standard error of the mean (SEM), with *n* = 4-7 animals per group. Statistical analysis, Two-way ANOVA followed by Tukey's *post hoc* test. *P < 0.05 vs. control group; ^ƒ^*P* < 0.05 vs. MRA and ethanol-MRA groups.

Eicosanoids derived from arachidonic acid may act as mediators of ethanol-induced tissue dysfunction. The impact of ethanol consumption on eicosanoid production was evaluated by measuring TXA₂ and PGE₂ levels. Increased levels of TXB_2_, a stable metabolite of TXA_2_, were detected in the left ventricle of ethanol-treated rats, and treatment with the MRA prevented this response (Figure 3A). However, no changes in TXB_2_ levels were found in the renal cortex after treatment with ethanol or the MRA (Figure 3B). No changes in PGE_2_ levels were detected in the left ventricle of ethanol-treated rats (Figure 3C). Conversely, increased concentrations of PGE_2_ were found in the renal cortex after treatment with ethanol, a response that was prevented by the MRA (Figure 3D). These results indicate that the effects of ethanol on eicosanoids production are tissue-specific and mediated by MR.

**Figure 4:**
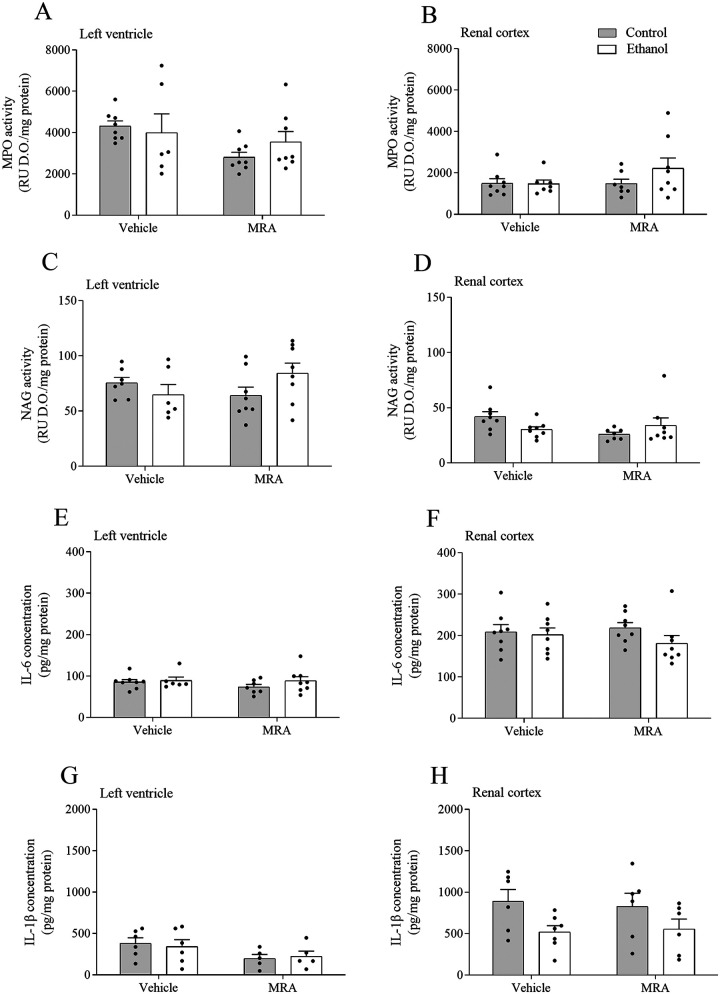
Biomarkers of inflammation in the left ventricle and the renal cortex of ethanol-treated rats. The activities of mieloperoxidase (MPO) **(A,B)** and N-acetyl-beta-D-glucosaminidase (NAG) **(C,D)** were determined colorimetrically as an estimative of neutrophils and macrophages infiltration. The concentrations of the pro-inflammatory cytokines interleukin (IL)-6 **(E,F)** and IL-1β **(G,H)** were determined by ELISA. Control, rats receiving vehicle treatment; Ethanol, rats exposed to ethanol; MRA, rats treated with mineralocorticoid receptor antagonist; Ethanol + MRA, rats co-treated with ethanol and MRA. Results are expressed as mean ± standard error of the mean (SEM), with *n* = 5-8 animals per group. Statistical analysis, Two-way ANOVA followed by Tukey's *post hoc* test.

Because ethanol consumption triggers pro-inflammatory responses that contribute to its harmful effects, we evaluated the possible role of inflammation in the early stages of ethanol-induced cardiorenal dysfunction by measuring inflammatory markers. Neither ethanol nor the MRA altered the activities of MPO (Figures 4A,B) or NAG (Figures 4C,D) in the left ventricle or the renal cortex. Similarly, no changes in the concentrations of IL-6 (Figures 4E,F) and IL-1β (Figures 4G,H) were found in the left ventricle or the renal cortex after treatment with either ethanol or the MRA, suggesting that, in the early stages of ethanol consumption, there is no activation of a pro–inflammatory response in the cardiorenal system.

## Discussion

Our results revealed that MR mediated the increase in O_2_•^−^ generation in the renal cortex and left ventricle of ethanol-treated rats. Blockade of MR prevented the increase in lucigenin luminescence in both tissues, suggesting that MR activation induces O_2_•^−^ generation through NADPH oxidase activation. However, it is important to note that O₂•⁻ is also generated by sources other than NADPH oxidase, including xanthine oxidase, uncoupled eNOS, and mitochondria. Therefore, although MR blockade may inhibit NADPH oxidase, our data do not allow us to conclude that the observed reduction in O₂•⁻ levels is specifically mediated via this pathway.

SOD converts O_2_•^−^ into H_2_O_2_, whose levels are controlled by catalase and GPx. Ethanol increased H_2_O_2_ levels in the renal cortex, but not in the left ventricle, showing that this effect is tissue-specific. The mechanism whereby this response occurs is unknow, but it seems not to involve an increase in SOD activity or a decrease in catalase or GPx activities, since no changes in these enzymes were evidenced in our study.

The overproduction of O_2_•^−^ is associated with lipoperoxidation, a process that results from the reaction of ROS, such as O_2_•^−^, with lipids of the cellular membrane. Lipid oxidation disrupts membrane integrity altering its permeability and, eventually, leading to cell death (24). Lipoperoxidation is one important mechanism by which ethanol promotes its deleterious effects, and NADPH oxidase-derived O_2_•^−^ plays a critical role in ethanol-induced lipid peroxidation in the cardiorenal system (1, 2, 4, 25). Our results confirm these previous findings and additionally show that MR are implicated in such response. The protective effect of the MRA against ethanol-induced lipoperoxidation probably results from its ability in reducing the overproduction of O_2_•^−^.

Despite the increase in ROS levels and lipoperoxidation, no changes in CK-MB activity were detected in our study. This result is consistent with previous findings showing that short exposition to ethanol increases myocardial ROS generation, but do not induce heart muscle injury (26, 27). The lack of effect of ethanol may be explained by the fact that increased oxidative stress is an early and initiating mechanism whereby ethanol consumption promotes cardiac dysfunction (28). Although it did not promote cardiac damage, ethanol consumption induced renal dysfunction, as evidenced by increased serum creatinine concentrations. The toxic effect of ethanol on the kidneys can be explained by the increased production of ROS and lipid peroxidation, which are described as two mechanisms associated with ethanol-induced kidney injury (1, 29). Therefore, it can be observed that ethanol differentially affects the cardiorenal system.

Ethanol promotes redox imbalance by reducing enzymatic and non-enzymatic antioxidant defenses (10). Importantly, the aldosterone/MR pathway has been shown to reduce the expression of antioxidant enzymes such as SOD and catalase (30). Thus, MR activation may contribute to the effects of ethanol on antioxidant systems. However, in the present study, we found that ethanol did not alter cardiac or renal antioxidant systems. The impact of ethanol on these systems is tissue–specific and depends on the duration of exposure. Accordingly, longer exposure periods (3–10 months) lead to reductions in antioxidant activity (31, 32).

In addition to oxidative stress, ethanol consumption triggers pro-inflammatory and immunomodulatory effects that ultimately alters tissue function. In this sense, it has been previously shown that ethanol leads to an increased production of prostanoids (e.g., TXA_2_ and PGE_2_) that mediate tissue dysfunction (13, 33). Increased TXA_2_ concentration was detect in the left ventricle, but not renal cortex of ethanol-treated rats. Ethanol is known to stimulate TXA_2_ production by increasing the expression of COX-2 and of thromboxane synthase, a key enzyme in the final step of TXA_2_ synthesis (33, 34). These mechanisms may help explains our present results showing that ethanol increases TXA_2_ concentration. A similar mechanism could help explain the increase in PGE_2_ levels in the kidney of ethanol-treated rats. In this sense, ethanol would increase the activity or expression of the isoforms of PGE_2_ synthase, leading to an increased generation of this prostanoid. This hypothesis is strengthened by the observation that ethanol consumption increases the production of PGE_2_ (35). Oxidative stress is associated with increased phospholipase A₂ activity and arachidonic acid availability (36). Thus, these responses may also explain the increase in eicosanoid levels described here. The blockade of MR fully prevented ethanol-induced increases in TXA_2_ and PGE_2_ levels, showing that these responses are mediated by MR.

Recruitment of immunological cells and augmented levels of pro-inflammatory cytokines are also mechanisms whereby ethanol promotes its harmful effects in some tissues (37). We found that ethanol consumption did not alter the activities of MPO or NAG in the left ventricle and renal cortex, suggesting no accumulation of neutrophils and macrophages in such tissues. Similarly, no changes in the concentration of IL-6 and IL-1β were found after treatment with ethanol. A possible explanation for such findings is that the effects of ethanol on pro-inflammatory parameters are dependent on the dose and period of treatment (38). Moreover, other aspects that should be considered are that the effects of ethanol on the concentration of pro-inflammatory cytokines are tissue- and species-specific (27, 39–42).

Some limitations of this study should be acknowledged. First, assessing cardiac and renal function, along with evaluating the effects of ethanol on tissue histology, would help clarify the impact of the molecular changes described herein and enhance the significance of our findings. Second, although serum creatinine is widely used as a marker of renal (dys) function, its levels can be influenced by muscle metabolism and catabolic states. Furthermore, creatinine typically rises only after established functional impairment, which limits its sensitivity for detecting early injury. The use of more sensitive markers of renal injury, such as kidney injury molecule 1, neutrophil gelatinase-associated lipocalin, or assessment of albuminuria/proteinuria, would strengthen the biological relevance of the observed oxidative alterations.

In conclusion, the MR pathway modulates renal dysfunction in response to ethanol and this effect is associated with increased production of O_2_•^−^, lipoperoxidation and the overproduction of PGE_2_. Ethanol is linked to the cardiac activation of MR, which drive the overproduction of O_2_•^−^, lipoperoxidation and increased TXA_2_ levels, but these molecular alterations are not related to cardiac damage. Ethanol consumption adversely affects the cardiorenal system, leading to tissue dysfunction and damage, which are associated with increased morbidity and mortality rates. Considerable progress has been made in understanding the mechanisms underlying ethanol-induced cardiorenal dysfunction. Identifying key molecular targets within the pathways of ethanol-associated tissue injury could facilitate the development of novel therapeutic agents aimed at preventing cardiorenal diseases resulting from ethanol consumption or improving the life expectancy of affected patients.

## Data Availability

The raw data supporting the conclusions of this article will be made available by the authors, without undue reservation.
